# *Tremella fuciformis* Polysaccharides Attenuate Oxidative Stress and Inflammation in Macrophages through miR-155

**DOI:** 10.1155/2018/5762371

**Published:** 2018-05-02

**Authors:** Yang Ruan, Hong Li, Lianmei Pu, Tao Shen, Zening Jin

**Affiliations:** ^1^Ward Thirty-Three, Department of Emergency Cardiology, Beijing Anzhen Hospital, Capital Medical University, Beijing 100029, China; ^2^The MOH Key Laboratory of Geriatrics, Beijing Hospital, National Center of Gerontology, Beijing 100730, China

## Abstract

**Aim:**

To investigate the function of *Tremella fuciformis* polysaccharides (TFPS) in LPS-induced inflammation and oxidative stress of macrophages.

**Methods:**

RAW264.7 cells were pretreated with TFPS and then stimulated with 0.1 *μ*g/ml LPS. NF*κ*B, Akt, p38MAPK, MCP-1, and SOD-1 were analyzed by Western blotting. Cell viability was measured using MTT assays. Reactive oxygen species (ROS) production, real-time PCR, ELISA, and immunofluorescence staining were performed on RAW264.7 cells that were treated with LPS and/or TFPS to investigate the anti-inflammatory effect of TFPS.

**Results:**

LPS induced inflammation and ROS production and promoted the secretion of cytokines such as TNF-*α* and IL-6. LPS also enhanced the nuclear translocation of NF*κ*B, which promoted inflammation by oxidative stress. However, pretreatment with TFPS profoundly inhibited the activation of Akt, p38MAPK, and NF*κ*B and attenuated the expression of MCP-1 in macrophages. Meanwhile, TFPS also decreased cytokine and ROS levels and attenuated cell inflammation after treatment with LPS. Moreover, miR-155, one of the key small RNAs which regulate NF*κ*B and inflammation in macrophages, was significantly downregulated.

**Conclusion:**

TFPS inhibits LPS-induced oxidative stress and inflammation by inhibiting miR-155 expression and NF*κ*B activation in macrophages, which suggests that TFPS may be a potential reagent for inhibiting the development of inflammation.

## 1. Introduction

Antioxidants in food and herbs may prevent free radical-induced cell damage and inflammatory reactions. Many natural plants and fungi contain antioxidant compounds, such as phenolic compounds, which can be used as antioxidants to alleviate the damage caused by free radicals and inflammation [1]. Polyphenols are important natural compounds that can be found in many vegetables, fruits, red wines, and cereals. Epidemiological studies have also revealed a negative correlation between the risk of clinical chronic diseases and the consumption of a polyphenol-rich diet [2].

In China, many herbs have been used in traditional Chinese medicine for centuries. *Tremella fuciformis*, a type of edible mushroom, exhibits various biological functions, including immunomodulatory [3], anticancer [4], hypolipidemic [5], and hypoglycemic [6] effects. Traditionally, *Tremella fuciformis* has been used as a dietary therapy, but knowledge of the pharmacological value of *Tremella fuciformis* is still limited. Thus, it is necessary to analyze the active ingredient of *Tremella fuciformis* for possible clinical applications. In recent years, many studies have suggested that the active ingredients of *Tremella fuciformis* are polysaccharides [7]. In addition, increasing studies have focused on the antioxidative and anti-inflammatory roles [8] of *Tremella fuciformis* polysaccharides (TFPS). Therefore, we performed a detailed investigation of the underlying mechanism of the antioxidative and anti-inflammatory roles of TFPS in our study.

Macrophages, which are a major contributor to inflammation, modulate inflammation and oxidation through Akt, p38MAPK, NF*κ*B, MCP-1, and SOD-1. The transcription factor NF*κ*B is an important regulator of the development of inflammation; when NF*κ*B is fully activated by antigens or inflammatory factors, NF*κ*B triggers a cytotoxic immune response. Furthermore, NF*κ*B can upregulate proinflammatory cytokines such as TNF-*α* and IL-6, which further enhance the inflammatory response [9]. In addition to its role in inflammation, NF*κ*B regulates other cellular processes [10], including cell proliferation and apoptosis. Therefore, NF*κ*B cannot be ignored in the process of exploring inflammation mediated by macrophages. The Akt signaling pathway is a classical inflammatory pathway that can modulate inflammation in macrophages. It has been reported that the Akt signaling pathway can modulate a variety of processes, such as growth, differentiation, apoptosis, and oxidative stress. MicroRNAs (miRNAs) are non-protein-encoding small RNAs which are negatively regulating target gene expression at the posttranscriptional level. Many studies have demonstrated that miRNA plays an important role in disease initiation and development by regulating distinct disease-related signal transduction pathways [11]. Recently, increasing evidence has indicated that several miRNAs act as key regulators of macrophage activation and inflammatory reaction [12]. It has been reported that miR-155 has an extensive and close association with NF*κ*B activation in macrophage. And many studies indicated that miR-155 acts as a key mediator of inflammation in atherosclerosis by repressing B-cell and promoting NF*κ*B activation in macrophages [12, 13].

TFPS, the polysaccharide fraction purified from *Tremella fuciformis*, has antioxidant and anti-inflammation properties [6]. However, little is known about the function of TFPS in the regulation of macrophage activation and initiation of inflammation. In our study, we examine the actions of TFPS in LPS-induced oxidative stress and inflammation in macrophages.

In this study, we investigated the protective effects of TFPS in LPS-induced oxidative stress and inflammation in macrophages and sought to gain novel insights into how TFPS regulates macrophage inflammation. Our results revealed that TFPS could attenuate free radical production and alleviate the inflammatory reaction by regulating the nuclear translocation of NF*κ*B. These findings provide insight into the mechanisms of TFPS in the regulation of macrophage inflammation and a new potential treatment for inflammation-related diseases.

## 2. Materials and Methods

### 2.1. Reagents

LPS was purchased from Sigma-Aldrich. Antibodies against phosphorylated-Akt, Akt, phosphorylated-p38MAPK, p38MAPK, phosphorylated-NF*κ*B, and NF*κ*B were purchased from Cell Signaling Technology. The MCP-1, SOD-1, and GAPDH antibodies were purchased from Santa Cruz Biotechnology. Secondary antibodies against rabbit or goat were purchased from Cell Signaling Technology. All other chemicals were purchased from Sigma or Amresco, unless specified otherwise.

#### 2.1.1. Preparation and Purification of TFPS

Single factor tests for hot water extraction of *Tremella fuciformis* polysaccharide were conducted with polysaccharide yield as the criteria index as well as solid to solvent ratio (g : ml), extraction time, and extraction temperature as the influencing factors. Based upon the test results, a response surface analysis was derived according to Box-Behnken central composite experimental design. The *Tremella fuciformis* dry fruiting body was ground into powder (40 mesh) using a food mixer. The optimal extraction conditions used were as follows: extraction temperature, 97.18°C; solid-liquid ratio, 1 : 60 g/ml; and extraction time, 6 h. The *Tremella fuciformis* dry fruiting powder was placed in a hot water bath shaker at 120 rpm for extraction and then centrifuged at 4000 rpm at 4°C for 20 min. The supernatant was precipitated with 98% ethanol, deproteinized by the Sevag method, purified through a dialysis membrane, and freeze-dried to obtain pure TFPS. Under the above conditions, the yield of *Tremella fuciformis* polysaccharide reaches 20.52% while the yield obtained by quantitative analysis is 20.25%, as reported previously [14].

### 2.2. Cell Culture and Transfection

RAW 264.7 cells were cultured in DMEM containing 10% fetal bovine serum, 2 mmol/l glutamine, and antibiotics (10 mol/l penicillin G and 10 mol/l streptomycin) at 37.0°C in a humidified atmosphere with 5% CO_2_. Chemically synthesized miR-155 mimics (mature sequence, UUA AUG CUA AUU GUG AUA GGG GU; complementary sequence, AAU UAC GAU UAA CAC UAU CCC CA) or negative control (NC) was transfected by Lipofectamine 2000 (Invitrogen, Carlsbad, CA, USA) according to the manufacturer's protocol. Eight hours after the transfection, the media were discarded and replaced with fresh medium. After additional 24-hour culture, the RAW 264.7 cells were treated with/without TPFS and then treated with LPS for indicated time [12].

### 2.3. Real-Time PCR Analysis of miRNA

Quantitative real-time PCR was performed using the iQ5 Optical System (Bio-Rad Laboratories) in combination with SYBR Green (Roche Applied Science, Mannheim, Germany) as described previously [15]. Briefly, total RNA was extracted using TRI Reagent (Sigma). 1.0 *μ*g of total RNA was then reverse-transcribed to the first-strand cDNA using Moloney murine leukemia virus reverse transcriptase (Promega Corp.) following the manufacturer's protocol. The primers used were as follows: miR155, forward primer: 5′-AATGCTAATTGTGATAGGGGT-3′, reverse primer was provided in the kit and U6, forward primer: 5′-CTCGCTTCGGCAGCACA-3′, reverse primer: 5′-AACGCTTCACGAATTTGCGT-3′. The real-time PCR profile was as follows: 95°C for 30 s and 40 cycles at 95°C for 30 s, 60°C for 30 s, and 72°C for 30 s. PCR conditions were the same as above, and relative expression was calculated by 2^−ΔΔCt^. The data presented are the average of four independent experiments.

### 2.4. Cell Viability Assay

MTT was used to measure cell viability, and the experiments were performed according to the manufacturer's instruction. RAW264.7 cells (5000 cells/well) were seeded into 96-well plates. All samples were analyzed in triplicate. In the TFPS group, the RAW264.7 cells were pretreated with TFPS before adding LPS. The cells in all wells were cultured in 0.5 mg/ml 3-[4,5-dimethylthiazol-2-yl]-2,5-diphenylterazolium bromide for 4 h, and the absorbance values at 490 nm were obtained, as described previously. The MTT kit was purchased from Roche Applied Science.

### 2.5. In Situ Detection of Reactive Oxygen Species (ROS) Production

RAW264.7 cells were stained with 10 *μ*M/l DHE (Sigma) for 30 min in a dark humidified chamber at 37.0°C to assess ROS production in situ. ROS production was detected with red fluorescence and visualized by fluorescence microscopy as described previously [15].

### 2.6. Detection of Inflammatory Factors by ELISA

We treated RAW264.7 cells with LPS (100 *μ*g/ml). Then, the cells were collected, and the cytokine concentrations were determined using ELISA kits (Invitrogen and R&D Systems). The IL-6 and TNF-*α* assessments were performed according to the manufacturer's instructions. The cytokine concentrations were normalized to the protein concentrations using a BCA protein assay (Pierce). Three independent experiments were performed. The data are expressed as the fold induction compared with the control (cells incubated with medium containing 0.1% DMSO) [16].

### 2.7. Western Blotting

The proteins were analyzed using SDS-PAGE and electro-transferred onto PVDF membranes. We blocked the membranes with 1% bovine serum albumin for 1 h and incubated the membranes with specific antibodies for 2 h. After five washes with TBST (0.1% Tween 20 in TBS), the membranes were incubated with horseradish peroxidase-conjugated secondary antibodies in TBST for 1 h. The bands were detected using chemiluminescence detection agents. Blot densitometry was performed, and the bands were analyzed using ImageJ software [16].

### 2.8. Immunofluorescence of NF*κ*B

RAW264.7 cells were grown on sterilized cover slips that were placed in a 6-well tissue culture plate. After treatments with LPS or LPS plus TFPS for 1 h, the cells were fixed in 4% (*w*/*v*) paraformaldehyde/PBS for 15 min. The cells were permeabilized with 1% Triton X-100/PBS for 15 min. Then, the cells were incubated with a blocking solution overnight at 4°C, and the anti-NF*κ*B p65 antibody was added for 2 h at 37°C. After washing, the cells were incubated with Alexa 488-conjugated anti-rabbit IgG antibody for 0.5 h at 37°C. Then, Hoechst 33342 was added for 20 min to stain the nuclei. NF*κ*B p65 was imaged using a fluorescence microscope (BX60; Olympus). NF*κ*B p65 was observed as green fluorescence, and the nucleus was observed as blue fluorescence [17].

### 2.9. Statistical Analyses

Data are presented as the mean ± SEM. One-way ANOVA followed by the Bonferroni procedure was used for multiple group statistical comparisons. *P* < 0.05 was considered statistically significant.

## 3. Results

### 3.1. LPS-Induced Inflammation and Oxidative Stress in Macrophages

**Figure 1:**
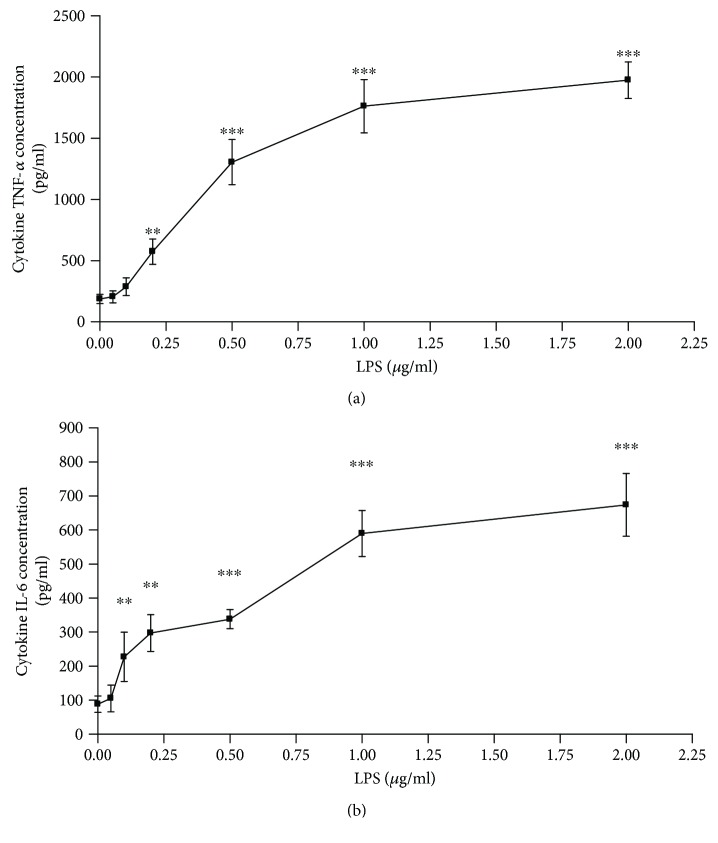
LPS promotes the release of inflammatory factors from RAW264.7 cells. (a) RAW264.7 cells were cultured in the presence of LPS (0, 0.05, 0.10, 0.20, 0.5, 1.0, or 2.0 *μ*g/ml) for 24 h. The concentrations of TNF-*α* were determined using ELISAs (*n* = 4). (b) RAW264.7 cells were cultured in the presence of LPS (0, 0.05, 0.10, 0.20, 0.5, 1.0, or 2.0 *μ*g/ml) for 24 h, and the concentrations of IL-6 were determined using ELISAs (*n* = 4, ^∗∗^*P* < 0.01, ^∗∗∗^*P* < 0.001 versus the control group).

**Figure 2:**
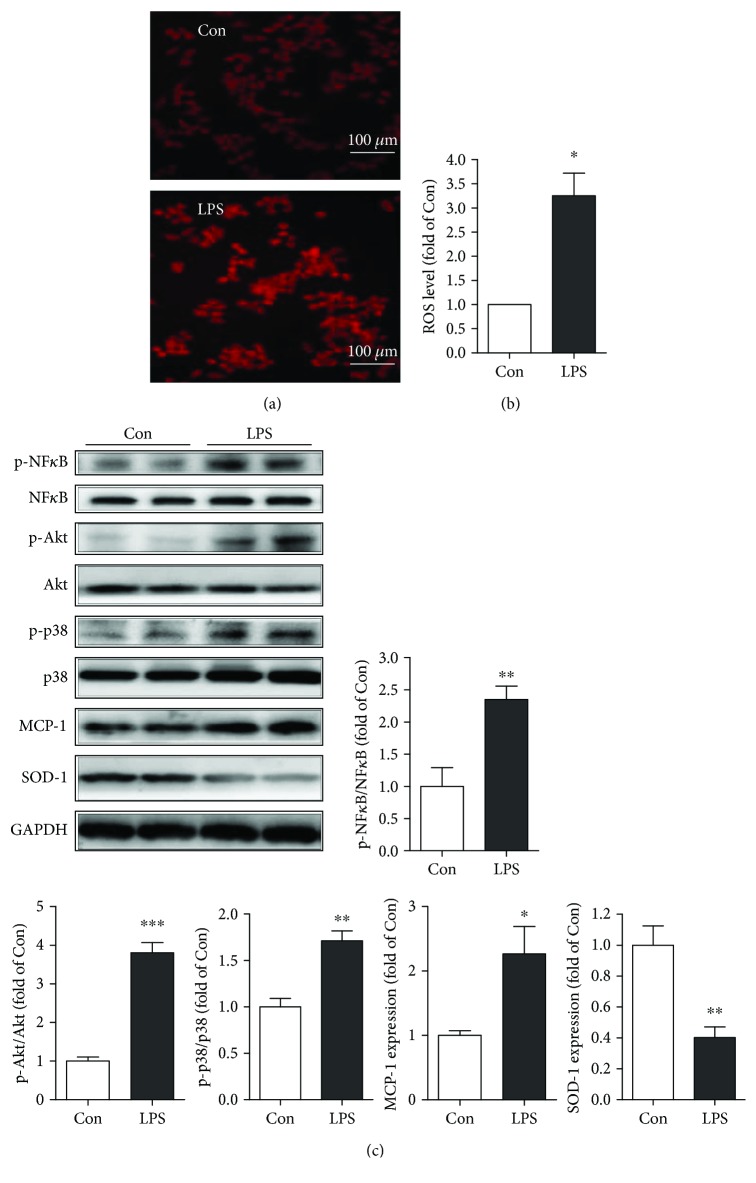
Oxidative stress-associated injury and the signaling pathways involved in LPS-induced inflammation of RAW264.7 cells. (a, b) DHE staining of the control and LPS pretreatment (1 *μ*g/ml) cells (*n* = 4, ^∗^*P* < 0.05 versus control). (c) Western blot analysis of the p-Akt, Akt, p-p38MAPK, p38MAPK, p-NF-*κ*B, NF*κ*B, MCP-1, SOD-1, and GAPDH protein expression levels in the control and LPS- (1 *μ*g/ml) pretreated (for 24 h) RAW254.7 cells (*n* = 4, ^∗^*P* < 0.05, ^∗∗^*P* < 0.01, ^∗∗∗^*P* < 0.001 versus control).

First, we developed a RAW264.7 cell inflammation model using different concentrations of LPS. As shown in Figures 1(a) and 1(b), RAW264.7 cells treated with 0–2 *μ*g/ml LPS produce TNF-*α* and IL-6 in a dose-dependent manner, and the cytokines reached their peak at 1 *μ*g/ml LPS. Therefore, we used 1 *μ*g/ml LPS to treat RAW264.7 cells for 12 h for the follow-up experiments. We also found that 1 *μ*g/ml LPS induced more reactive oxygen species (ROS) compared with the control RAW264.7 cells (Figures 2(a) and 2(b)). Moreover, we observed that LPS increased MCP-1 expression and decreased the expression of antioxidative stress protein SOD-1. Additionally, the proinflammatory signaling pathways, namely, Akt, p38MAPK, and NF*κ*B, were dramatically activated after the LPS treatment in Raw264.7 cells.

### 3.2. Cytotoxicity Analysis of TFPS in RAW264.7 Cells

**Figure 3:**
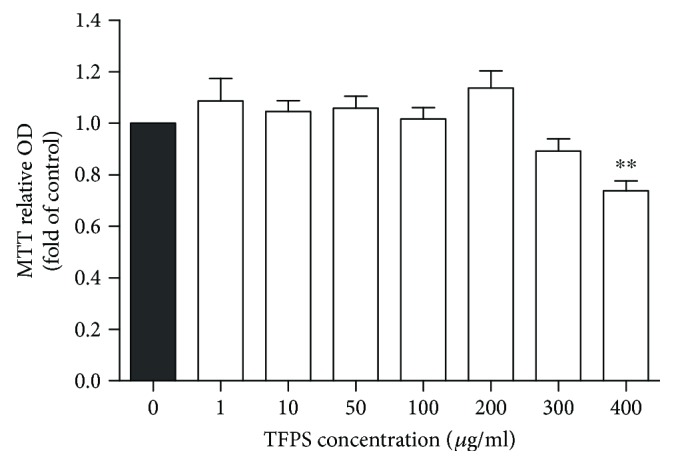
Cytotoxicity of *Tremella fuciformis* polysaccharide in RAW264.7 cells. RAW264.7 cells were cultured in the presence of TFPS (0, 10, 50, 100, 200, 300, and 400 *μ*g/ml) for 24 h; cell viability was assessed using MTT assays (*n* = 3, ^∗∗^*P* < 0.01 versus the control group).

To assess the potential function and safety of TFPS, we performed cytotoxicity analyses using MTT assays in RAW264.7 cells treated with various concentrations of TFPS. As shown in Figure 3, treatment with 0–300 *μ*g/ml TFPS for 48 h did not alter RAW264.7 cell viability.

### 3.3. TFPS Attenuates the Macrophage Inflammatory Reaction Induced by LPS

**Figure 4:**
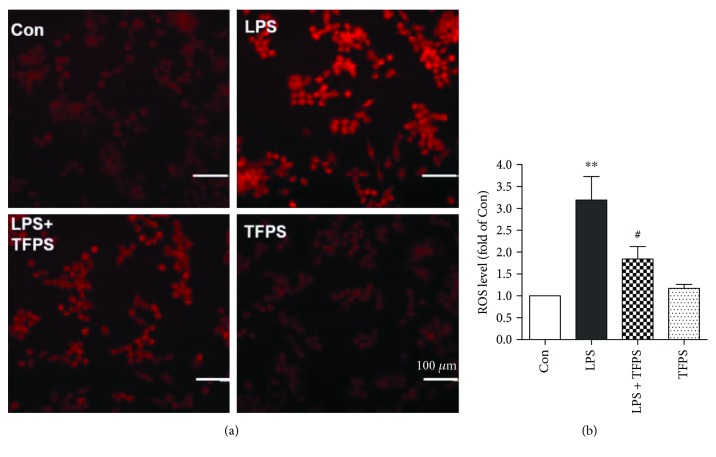
TFPS relieves LPS-induced RAW264.7 cell injury by attenuating oxidative stress. (a) DHE staining of control, LPS-treated (1 *μ*g/ml), TFPS-treated (200 *μ*g/ml), and TFPS-pretreated (200 *μ*g/ml) plus 1 *μ*g/ml LPS-treated (LPS + TFPS) cells. (b) The average levels of the ROS fluorescence intensity (*n* = 4, ^∗∗^*P* < 0.01 versus control; ^#^*P* < 0.05 versus the LPS + TFPS group).

LPS can cause macrophage inflammation. TNF-*α* and IL-6 are inflammatory cytokines that are present during macrophage activation and inflammation. We examined whether TFPS pretreatment could attenuate the LPS-induced cell inflammation reaction in macrophages. We used 0–300 *μ*g/ml TFPS to treat the RAW264.7 cells before the LPS incubation. The results showed that the TNF-*α* and IL-6 expression levels in RAW264.7 cells decreased in a TFPS concentration-dependent manner (Figure 4). And the optimal TFPS anti-inflammatory concentration is 200 *μ*g/ml. Therefore, we used 200 *μ*g/ml TFPS to treat RAW264.7 cells in follow-up experiments.

### 3.4. TFPS Inhibits Oxidative Stress in LPS-Treated Macrophages

**Figure 5:**
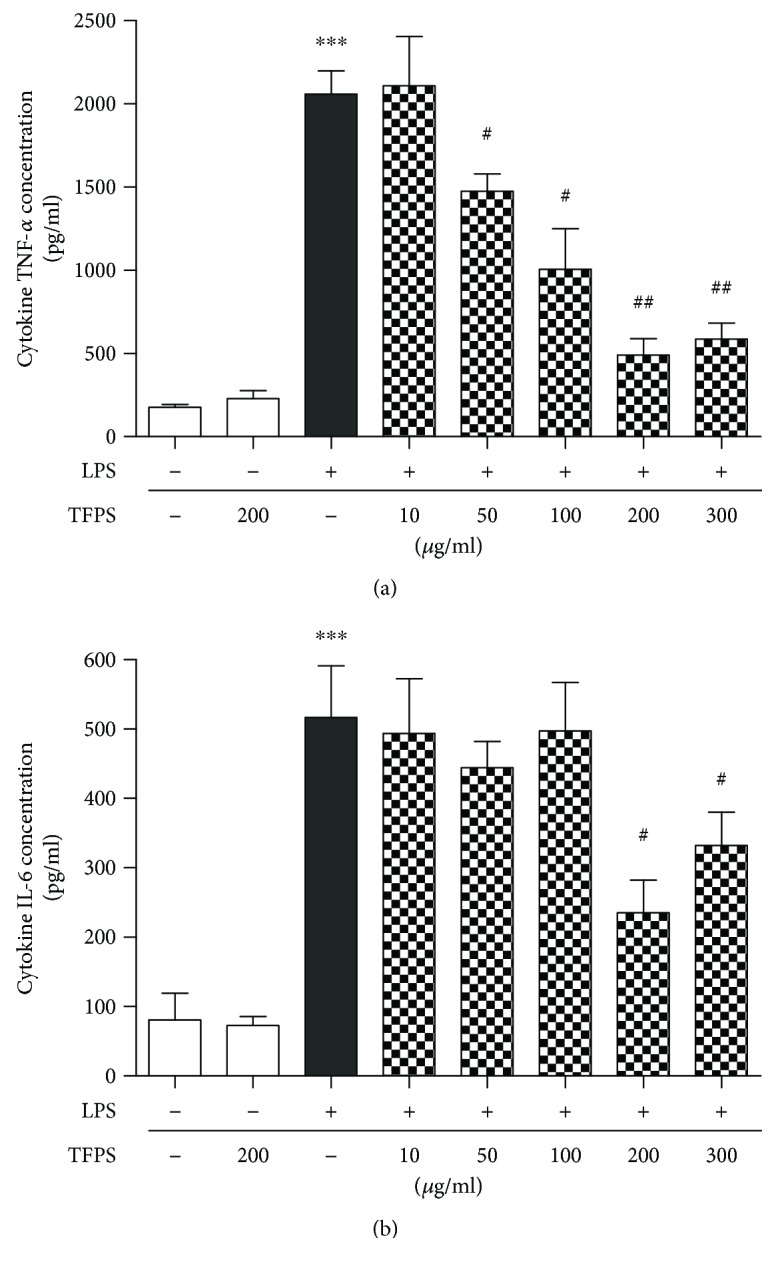
TFPS decreases the release of inflammatory factors that are produced during LPS-induced RAW264.7 cell injury. (a, b) ELISAs of TNF-*α* and IL-6 in control, TFPS-treated (200 *μ*g/ml), LPS-treated (1 *μ*g/ml), and 1 *μ*g/ml LPS-treated cells following TFPS pretreatments (10, 50, 100, 200, or 300 *μ*g/ml) of RAW264.7 cells for 1 h (*n* = 3, ^∗∗∗^*P* < 0.001 versus control; ^#^*P* < 0.05, ^##^*P* < 0.01 versus the LPS group).

LPS participates in the activation of RAW264.7 cells and promotes ROS production (Figure 5). Interestingly, pretreatment with TFPS effectively blocked ROS production in RAW264.7 cells after the LPS treatment (Figure 5). The data suggest that TFPS may attenuate macrophage inflammation by inhibiting ROS production and oxidative stress.

### 3.5. TFPS Decreases the Inflammatory Response of Macrophages by Inhibiting the Expression of MCP-1

**Figure 6:**
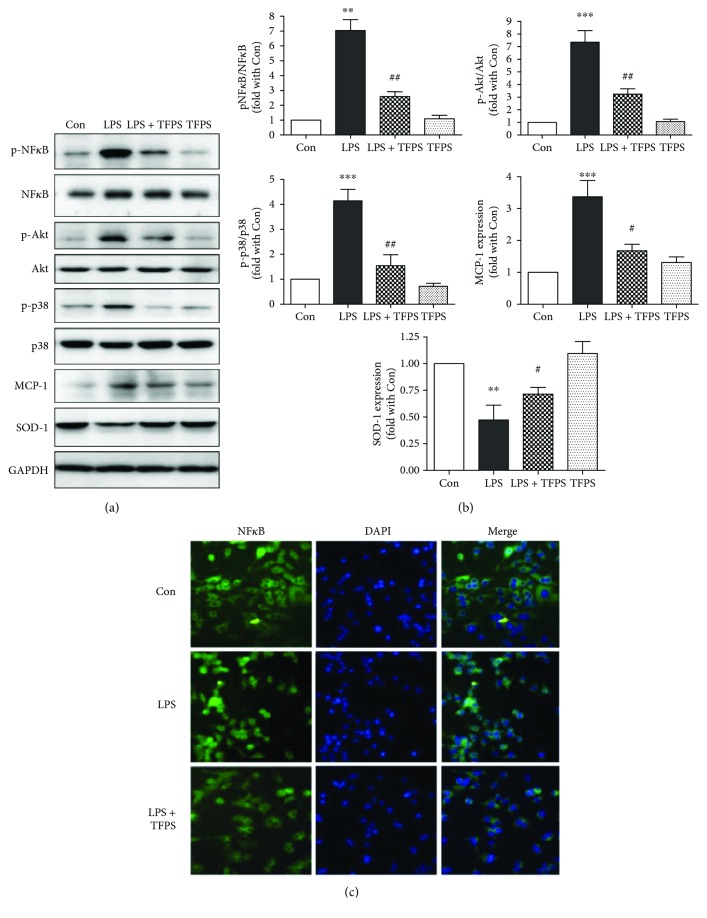
The signaling pathways that TFPS attenuates during LPS-induced RAW264.7 cell injury. (a, b) The phosphorylated-Akt, Akt, phosphorylated-p38MAPK, p38MAPK, phosphorylated-NF*κ*B, NF*κ*B, MCP-1, SOD-1, and GAPDH protein expression levels in control, LPS-treated (1 *μ*g/ml), TFPS-treated (200 *μ*g/ml), and TFPS-pretreated (200 *μ*g/ml) + 1 *μ*g/ml LPS-treated (LPS + TFPS) cells, as assessed by Western blotting (*n* = 4, ^∗∗^*P* < 0.01, ^∗∗∗^*P* < 0.001 versus control; ^#^*P* < 0.05, ^##^*P* < 0.01 versus the LPS + TFPS group). (c) Nuclear translocation of NF*κ*B in control, LPS-treated, and LPS + TFPS-treated RAW24.7 cells was analyzed by immunofluorescence microscopy using the NF*κ*B p65 subunit antibody (Green). The nuclei were stained with DAPI. Representative images of three independent experiments are shown.

To further investigate the relationship between TFPS and inflammation, we analyzed the expression of MCP-1 in macrophages. The Western blot results showed that LPS could induce macrophage inflammation by promoting the expression of MCP-1, which could be reversed by pretreatment with TFPS (Figure 6).

### 3.6. TFPS Inhibits the Inflammatory Response of Macrophages by Inhibiting Activation of NF*κ*B

It is well known that transcription factors such as nuclear factor *κ*B (NF*κ*B) play pivotal roles in the inflammatory and immune responses of macrophages. In our study, we found that TFPS reduced the inflammatory response of RAW264.7 cells by inhibiting phosphorylation of NF*κ*B (Figures 6(a) and 6(b)) after the LPS treatment. In addition, pretreatment with 200 *μ*g/ml TFPS reduced phosphorylation of NF*κ*B significantly. The immunofluorescence data also revealed the translocation of NF*κ*B from the cytoplasm to the nucleus in RAW24.7 cells after the LPS treatment (Figure 6(c)). In addition, TFPS reversed this process effectively to attenuate the inflammatory reaction after the LPS treatment.

### 3.7. TFPS Regulates Macrophage Activation and NF*κ*B Activity by Attenuating miR-155 Expression

**Figure 7:**
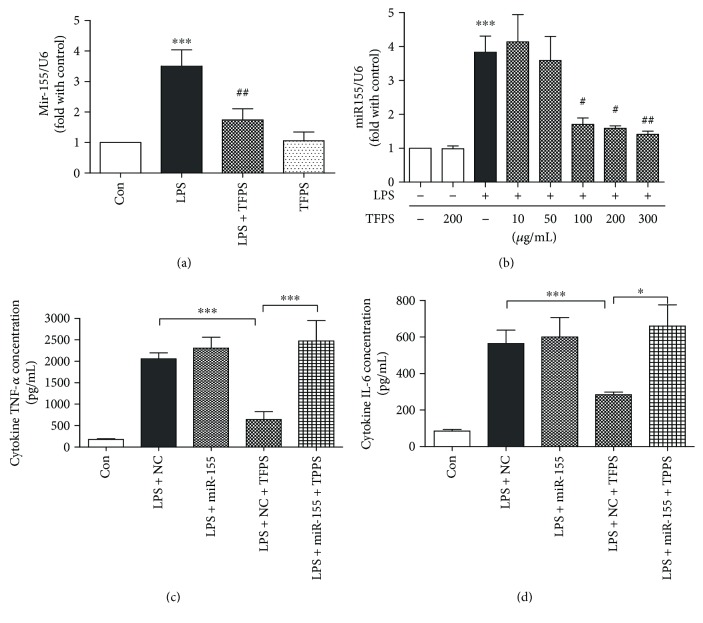
TFPS attenuates LPS-induced inflammation reaction by inhibition of miR-155. (a) The miR-155 expression levels in control, LPS-treated (1 *μ*g/ml), TFPS-treated (200 *μ*g/ml), and TFPS-pretreated (200 *μ*g/ml) + 1 *μ*g/ml LPS-treated (LPS + TFPS) cells, as assessed by real-time RT-qPCR (*n* = 4, ^∗∗∗^*P* < 0.001 versus control; ^##^*P* < 0.01 versus the LPS + TFPS group). (b) The miR-155 expression levels in control, TFPS-treated (200 *μ*g/ml), LPS-treated (1 *μ*g/ml), and 1 *μ*g/ml LPS-treated cells following TFPS pretreatments (10, 50, 100, 200, or 300 *μ*g/ml) of RAW264.7 cells for 1 h (*n* = 3, ^∗∗∗^*P* < 0.001 versus control; ^#^*P* < 0.05, ^##^*P* < 0.01 versus the LPS group). (c) ELISAs of TNF-*α* and IL-6 in control, LPS + NC, LPS + miR-155, LPS + NC + TFPS, and LPS + miR-155 + TFPS groups (*n* = 4, ^∗^*P* < 0.05, ^∗∗∗^*P* < 0.001).

We checked several important miRNAs which regulate macrophage inflammation in macrophages. We found that miR-155 displays an obvious increase during the inflammatory response to LPS, indicating the crucial role of miR-155 in the initiation of inflammation in macrophage (Figure 7(a)). In addition, we observed that TPFS sufficiently inhibited the upregulation of miR-155 in a dose-dependent manner (Figure 7(b)). And the protective effect could be blocked by miR-155 overexpression by Lipofectamine 2000. To better understand miR-155 function in the regulation of the macrophage acute inflammatory response, we checked TNF-*α* and IL-6 expression after the overexpression of miR-155 in macrophage by ELISA assay. miR-155 overexpression could sufficiently block the TNF-*α* and IL-6 expression inhibition effect of TFPS in macrophage after LPS treatments. Our data indicated that overexpression of miR-155 could sufficiently block the protective effect of TFPS and promote the activation of the macrophage and acute inflammatory effect.

## 4. Discussion

### 4.1. Determining the Safety of *Tremella fuciformis* Polysaccharides and Optimal Inflammatory Concentration in RAW264.7 Cells

In our study, we tested the cytotoxicity of TFPS in RAW264.7 cells. Our data indicated that at concentrations between 0 and 300 *μ*g/ml, there was no obvious cytotoxicity in RAW264.7 cells that were pretreated with TFPS, which suggested that the medicinal properties of TFPS were safe. Our data also indicates that the TNF-*α* and IL-6 expression levels in RAW264.7 cells decreased in a TFPS concentration-dependent manner. And the optimal TFPS anti-inflammatory concentration is 200 *μ*g/ml.

### 4.2. *Tremella fuciformis* Polysaccharides and Inflammation

*Tremella fuciformis* polysaccharides (TFPS), which are extracted from edible mushrooms, exhibit numerous biological functions, including immunomodulatory, antineoplastic, and hypoglycemic effects [7, 14, 18]. In our study, we measured the inflammation and oxidative stress of macrophages in response to TFPS. Inflammation, which is involved in various diseases, including heart disease [19, 20], metabolic disease [21], and tumors [22], has been well studied. Various components that are involved in the inflammatory network influence and interact with each other; for example, TNF-*α* and ROS, TNF-*α* and NF*κ*B, NF*κ*B and Akt (also known as PKB), p38MAPK and NF*κ*B, and ROS and MCP-1 are pairs that affect one another. Together, these factors promote the occurrence and development of inflammation. In our study, we explored the mechanism of the anti-inflammation activity of TFPS, as described above.

### 4.3. *Tremella fuciformis* Polysaccharides and Oxidative Stress

To further elucidate the function of TFPS in attenuating LPS-induced macrophage activation and inflammation, we checked LPS-induced cell oxidative stress treated with/without TFPS. Reactive oxygen species (ROS), which are continuously produced by cellular respiration and metabolism [23], are important for cellular functions. However, high concentrations of ROS, such as those observed during aging and inflammation, are harmful to the body [23, 24]. Oxidative stress in macrophages plays an important role in the initiation and progression of inflammation [24]. Inflammation can be inhibited by decreasing oxidative stress in macrophages. In our study, we found that TFPS could reduce oxidative stress in LPS-induced macrophages and inhibit its activation and inflammation reaction.

### 4.4. TFPS and NF*κ*B Activation

NF*κ*B is a family of five transcription factors that form homodimers or heterodimers to regulate the expression of cytokines and other immune-response genes. Many studies also indicated that nuclear NF*κ*B is not only involved in regulating adaptive immunity but is also important for the innate immune response [25]. Therefore, dysregulation of NF*κ*B causes inflammation [9, 26], cancer [9], and atherosclerosis [17]. It has been verified that inflammatory factors can promote NF*κ*B activity [9], and active NF*κ*B can also promote the expression of inflammatory factors [9]. Therefore, we examined the function of NF*κ*B in LPS-treated macrophages. Macrophage responses to pathogens are controlled by the activation p38MAPK and Akt, which are specifically connected to NF*κ*B activation. Our data indicated that TFPS could effectively block LPS-induced p38MAPK and Akt phosphorylation and attenuate NF*κ*B activation and nuclear translocation.

### 4.5. TFPS Inhibit LPS-Induced Macrophage Activation Mainly by the Downregulating miR-155 Expression

We have also shown that TFPS could mediate its anti-inflammatory role by altering miR-155 expression. Many studies indicated that miR-155 is one of the inflammation-related miRNAs by upregulation of NF*κ*B expression and activation [13]. In our study, LPS increased miR-155 expressions in RAW264.7 cells. Upregulation of miR-155 by LPS treatment was responsible for the abnormal inflammatory response development macrophage-induced injury in many organs and cells. Further, our study revealed that the anti-inflammatory action of TFPS on NF*κ*B-mediated inflammation reaction was partially associated with its downregulation of miR-155 in macrophage. And overexpression of miR-155 could reverse the protective effect of TPFS and the expression of TNF-*α* and IL-6. Thus, TFPS may inhibit the expression levels of NF*κ*B by targeting their regulatory miR-155 in macrophage through a positive feedback. In our study, we observed the upregulation of miR-155 in LPS-treated RAW264.7 cells, accompanied with the upregulation of NF*κ*B activation. Pretreatment of TFPS decreased miR-155 expression and inhibited NF*κ*B activation thus attenuated the inflammation reaction in macrophages.

Therefore, we presumed that TFPS could decrease inflammation by inhibiting the expression of miR-155 in LPS-treated macrophages, which implied that TFPS not only reduced the initiation of the inflammatory response but also inhibited the activation and augmentation of NF*κ*B signal pathway.

## 5. Conclusions

In summary, we have demonstrated that TFPS exhibits antioxidative stress and anti-inflammatory properties in LPS-treated macrophages by inhibiting the miR-155 and NF*κ*B pathways, suggesting that TFPS may be a potential therapeutic agent for the treatment of inflammation-related diseases.

## Additional Points

*Significance*. Our data show that TFPS can effectively inhibit LPS-induced macrophage activation and inflammation reaction. TFPS pretreatment prevents LPS-induced oxidative stress and inflammation in macrophages by inhibiting miR-155 expression and NF*κ*B activation and nuclear translocation. The authors conclude that TFPS can be used as a potential therapeutic agent for the treatment of oxidative stress- and inflammation-related diseases.
